# Effects of Milk or Soy Milk Combined with Mild Resistance Exercise on the Muscle Mass and Muscle Strength in Very Old Nursing Home Residents with Sarcopenia

**DOI:** 10.3390/foods10112581

**Published:** 2021-10-26

**Authors:** Feng-YI Chiang, Jiun-Rong Chen, Wei-Ju Lee, Suh-Ching Yang

**Affiliations:** 1 School of Nutrition and Health Sciences, College of Nutrition, Taipei Medical University, Taipei 11041, Taiwan; cute_pipi0621@yahoo.com.tw (F.-Y.C.); syunei@tmu.edu.tw (J.-R.C.); 2 Yuanshan Branch, Taipei Veterans General Hospital, Taipei 11217, Taiwan; 3 Aging and Health Research Center, National Yang Ming Chiao Tung University, Taipei 30010, Taiwan; leewju@gmail.com; 4 Department of Family Medicine, Yuanshan Branch, Taipei Veterans General Hospital, Taipei 11217, Taiwan; 5 Research Center of Geriatric Nutrition, College of Nutrition, Taipei Medical University, Taipei 11041, Taiwan; 6 School of Gerontology Health Management, College of Nursing, Taipei Medical University, Taipei 11041, Taiwan; 7 Nutrition Research Center, Taipei Medical University Hospital, Taipei 11041, Taiwan

**Keywords:** sarcopenia, milk, soy milk, mild resistance exercise, elderly subjects

## Abstract

**Background and aims:** Sarcopenia is recognized as a major public health issue, because it is prevalent in the elderly, especially those who live in long-term care facilities. The purpose of this study was to investigate the beneficial effects of milk or soy milk combined with resistance exercise on the muscle mass and muscle strength of individual elderly nursing home residents with sarcopenia. **Methods:** This study was a randomized controlled trial (clincaltrials.gov as NCT05035121) that recruited very old (>75 years) subjects with sarcopenia in a nursing home (Su-Ao and Yuanshan Branches, Taipei Veterans General Hospital) from June to December 2017. Thirty-five elderly (84.9 ± 6.1 years old) subjects were recruited and divided into three groups: control (*n* = 12), milk supplemented (*n* = 12), and soy milk supplemented (*n* = 11). All participants joined a mild resistance exercise training program three times a week (30 min/time). Moreover, elderly subjects in the milk and soy milk groups drank 200 mL of milk or soy milk for breakfast and as a snack after exercise. **Results:** After 12 weeks, compared to the baseline, calf circumferences had significantly increased in the control and soy milk groups (*p* = 0.0362 and *p* = 0.0197, respectively). Hand grip strength had significantly improved in the milk and soy milk groups (*p* = 0.0407 and *p* = 0.0096, respectively). In addition, there was no difference among the three groups. **Conclusions:** Mild resistance exercise combined with milk or soy milk improved the calf circumference and hand grip strength in very old nursing home residents with sarcopenia.

## 1. Introduction

Sarcopenia is one of the most important health problems in the elderly, which is defined as age-related declines in muscle mass and function [1]. The prevalence of sarcopenia in the elderly is around 10% worldwide [1]. In Taiwan, the situation of sarcopenia in the elderly is more serious as indicated by a higher prevalence (18.6% in females and 23.6% in males) [2]. According to a systematic review, the prevalence of sarcopenia was 14–33% in long-term care populations which was higher compared to community-dwelling populations and acute-care hospital populations [3].

The European Society for Clinical and Metabolism (ESPEN) suggests that the daily dietary recommended intake of protein is 1.0–1.2 g/kg body weight (BW) for healthy elderly adults; however, the intake of protein has to be increased to 1.2–1.5 g/kg BW for elderly adults with malnutrition or those at high risk of malnutrition [4]. The ESPEN also indicates that daily physical activity or exercise (resistance training, aerobic exercise) should be undertaken by all older people for as long as possible [4]. On the other hand, a number of trials examined the separate effects of increased exercise or dietary supplementation on muscle mass and physical performance of older adults, but the findings are inconsistent because of the different types, doses, frequencies, and delivery methods (provided at the same time as the exercise or not) across studies, and participants ranged in status from healthy older adults to those who were frail or sarcopenic [5,6]. Moreover, there have been few sarcopenia-related studies that focused on very old individuals.

More studies are needed to establish the role of the types of protein and amino acids, such as drinks, powder, etc., and whether there are important additional benefits for vulnerable older adult populations whose habitual protein intake is low. Denison et al. indicated that supplementation of protein such as milk, whey protein, or branched-chain amino acids, especially leucine, increased muscle strength, muscle mass, and physical function in old people [7]. However, for older individuals who live in a nursing home or long-term care facility, it is hard to get non-natural food nutritional supplementation. Milk is a common natural food that is used for protein supplementation due to the high content of leucine and the fact that it can easily be obtained from supermarkets. For the vegan, soymilk is used to replace milk as the dietary protein source. Therefore, using milk or soy milk as protein supplementation for the elderly is an ideal and economical nutritional strategy.

Therefore, we hypothesized that milk or soy milk supplementation combined with resistance exercise could increase muscle mass and improve muscle strength in very old individual nursing home residents with sarcopenia. This study was carried out to verify this hypothesis.

## 2. Materials and Methods

### 2.1. Study Design and Population

The study was approved by the Taipei Medical University Joint Institutional Review Board (TMU-JIRB No: N201702034, ClinicalTrails.gov ID: NCT05035121). All procedures were conducted according to the principles expressed in the Declaration of Helsinki.

Recruitment was conducted in two nursing homes, including the Yuanshan Branch and Suao Branch of Taipei Veterans General Hospital, from June to December 2017. Participants aged more than 75 years received a sarcopenia diagnosis based on the definition announced by The Asian Working Group for Sarcopenia (AWGS) [8]. Participants with sarcopenia were excluded if they had a milk or soy milk allergy, or they were unable to stand the resistance training exercise. Cancer, chronic liver disease, and kidney dialysis were also listed as exclusion criteria.

**Figure 1:**
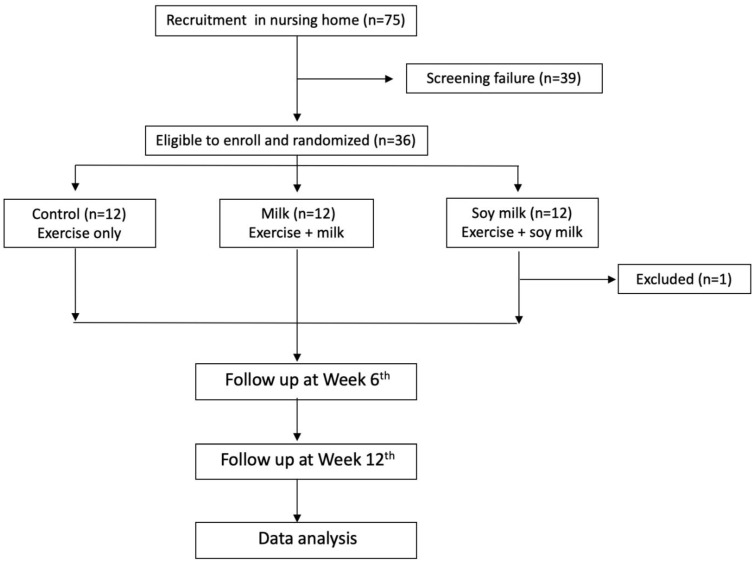
Study flow diagram for enrolling very old (>75 years of age) subjects with sarcopenia in a nursing home from June to December 2017. Thirty-six elderly were recruited and divided into three groups: control (*n* = 12), milk (*n* = 12), and soy milk (*n* = 12) groups. However, one subject (*n* = 1) was excluded from soy milk group due to bellyache after drinking soy milk. All participants joined a resistance exercise training program three times per week (30 min/time). Elderly subjects in the milk and soy milk groups drank 200 mL of milk or soy milk for breakfast and as a snack after exercise.

This study was a randomized controlled trial. Participants with sarcopenia were divided into three groups: control, milk, and soy milk groups. All of the groups underwent a mild resistance exercise training program for 12 weeks (3 times/week, 30 min/time), including chair exercise, resistance exercise with sandbags and elastic bands, and balance and gait training. Participants in the milk and soy milk groups drank 200 mL milk (96 kcal, 5.2 g protein, 2.6 g fat, 12 g carbohydrates, 260 mg Ca, 620.9 mg leucine) or soy milk (97.4 kcal, 6.4 g protein, 3.0 g fat, 11.2 g carbohydrates, 220 mg Ca, 458.6 mg leucine) for breakfast and an afternoon snack every day for 12 weeks (400 mL milk or soy milk/day). When there was exercise in the afternoon, milk, or soy milk was given after exercise. Meanwhile, participants of the control group were provided with 200 mL water. A study flowchart is presented in Figure 1.

### 2.2. Data Collection

#### 2.2.1. Sociodemographic Data

Age, sex, and medical history were collected to evaluate the current use of medications.

#### 2.2.2. Anthropometric, Clinical, and Laboratory Data

Anthropometric, clinical, and laboratory data were collected at the beginning and end of the experiment. Body-mass index (BMI; kg/m^2^) was calculated based on body weight and height. Moreover, dual X-ray absorptiometry (Inbody S10, Inbody Inc., Seoul, South Korea) was used to evaluate fat mass and lean mass. Appendicular skeletal muscle mass (ASM) was calculated as the sum of the lean mass of the limbs and normalized in relation to the subject’s height (ASM/height in meters squared) to obtain the ASM index. The measurements of clinical data were performed on blood samples collected in a fasted (>8 h) condition. Activities of alanine aminotransferase (ALT) as a liver function indicator and the creatinine level as a kidney function biomarker were analyzed by spectrophotometry (Beckman AU680, Beckman Coulter, Brea, CA, USA). Prealbumin level as a nutritional status indicator and high-sensitivity C-reactive protein (hsCRP) as an inflammatory indicator were measured with an automated clinical chemical analyzer (ADVIA 2400, Siemens Healthcare, Erlangen, Germany). Vitamin D status was represented by the 25-hydroxyvitamin D level which was measured by the Diasorin XL assay (DiaSorin, Saluggia, Italy). Insulin resistance (IR) indicators were also determined in this study, including the fasting blood glucose level, insulin level (Beckman AU680, Beckman Coulter), and glycated hemoglobin (HbA1c; by cation-exchange high-performance liquid chromatography (HPLC), Tosoh Europe, Tessenderlo, Belgium). The homeostasis model assessment of IR index (HOMA-IR) was calculated according to the formula: fasting insulin (μU/mL) × fasting glucose (mmol/L)/22.5. Insulin-like growth factor (IGF)-1, which is considered to be related to protein synthesis in muscles [9], was measured by a chemiluminescence immunoassay (Immulite2000, Siemens Healthcare).

#### 2.2.3. Sarcopenic Index

Muscle mass and body fat were evaluated by a bioelectrical impedance analysis (BIA) (Inbody S10, Inbody, Seoul, South Korea), which was used to predict the appendicular skeletal muscle mass (ASM) which was divided by height squared (m^2^) according to the formula of Kim et al. [10]. The calf circumference was measured with a measuring tape around the thickest point of the calf with the tape stuck tightly without squeezing the skin. The muscle strength was evaluated by the hand grip strength using a Smedley dynamometer (TTM-YD, Tsutsumi Industries, Tokyo, Japan). The gait speed test was performed by recording the average time of walking 6 m and is presented as the distance per second (m/s).

#### 2.2.4. Diet Assessment

Dietary data were collected based on the meal menu of the nursing home including one weekday and two weekends at the beginning and the end of the experiment. Caregivers recorded the actual food intake by participants. All data were analyzed with Ekitchen Nutrients Analysis Software (E kitchen Business, Taichung, Taiwan) based on the food and nutrient database created by the Organization Act of the Food and Drug Administration, Ministry of Health and Welfare, Taiwan.

### 2.3. Statistical Analysis

Statistical analyses were performed with SAS vers. 9.4 (SAS Institute, Cary, NC, USA), and all values are expressed as the mean ± standard deviation (SD), or number (*n*) and percentage (%). A paired *t*-test was used when comparing differences after the intervention within a group. A one-way analysis of variance (ANOVA) with a post hoc Tukey’s test was used to compare the differences in basic characteristics among groups. The effects of two factors, such as time and nutritional treatment, were assessed by using a two-way ANOVA with a post hoc Tukey’s test. Chi-squared tests were used to examine differences between categorical variables. A *p* value of <0.05 was considered statistically significant.

## 3. Results

### 3.1. General Characteristics and Clinical Data at the Baseline

A total of 35 participants, including 29 males and 6 females, completed all evaluations in this study (Figure 1). The average age was 84.97 ± 6.17 (78–93) years old. Around 20% of participants were older than 90 years. There were no differences among the three groups in general characteristics or in clinical and laboratory data at the baseline (Table 1 and Table 2). All of the groups showed normal ALT activity, creatinine levels, and prealbumin levels (Table 2). However, insufficient 25-hydroxyvitamin D was found in all groups according to the reference range in Holick’s study [11]. In addition, blood hsCRP levels were higher in all groups at week 12 based on the low risk of heart disease (<1.0 mg/dL) [12,13]. Although fasting blood sugar and insulin levels were still in the normal range, higher HbA1c levels (>5.6%) were found in all groups [14]. The level of IGF-1 was slightly lower in the control group according to the reference range reported by Bidlingmaier et al. [15]. After 12 weeks, no changes were observed; however, blood 25-hydroxyvitamin D levels still were insufficient in all groups (Table 2). Moreover, the blood IGF-1 level was significantly elevated in the soy milk group when compared with that of the control group (*p* = 0.0023).

### 3.2. Anthropometric Data and the Sarcopenic Index

There were no changes in the BW, BMI, or body fat during the experimental period (Table 3). In addition, no differences were found among the three groups in anthropometric data (Table 3). However, all groups had a high body fat percentage as defined by the Health Promotion Administration, Ministry of Health and Welfare, Taiwan [16].

When compared to the baseline, calf circumferences significantly increased in the control (*p =* 0.0362) and soy milk (*p =* 0.0197) groups (Table 3) after 12 weeks. Hand grip strength also improved in the milk (*p* = 0.0407) and soy milk (*p* = 0.0096) groups at week 12 compared to the baseline (Table 2). Regarding gait speed, only the control group showed a significantly higher value after 12 weeks (*p* = 0.04, Table 2). However, no obvious difference was observed in the sarcopenic index among the three groups (Table 3).

According to The European Working Group on Sarcopenia in Older People (EWGSOP), sarcopenia can be classified into three stages: pre-sarcopenia, sarcopenia, and severe sarcopenia [17]. In this study, participants with sarcopenia were also classified into three stages. As shown in Table 4, the number of participants with severe sarcopenia decreased in all groups.

### 3.3. Dietary Intake

The daily dietary intake which did not include the intake of milk or soy milk is shown in Table 5. All groups had similar consumptions of total calories, carbohydrates, protein, and fat at the baseline and also at week 12. Due to nutritional supplementation, protein intake was increased from 1.3 to 1.5 g/kg BW in the milk group and from 1.4 to 1.6 g/kg BW in the soy milk group.

## 4. Discussion

### 4.1. Nutritional Status and Inflammation Index in Nursing Home Residents

In this study, no malnutrition problem occurred in participants, except low blood levels of 25-hydroxyvitamin D. Chen et al. also indicated that prevalence of vitamin D insufficiency and deficiency in community-living older adults (Ilan City, Taiwan) were 50.5% and 33.6%, respectively [18]. Risk factors for a vitamin D deficiency include older age, malnutrition, obesity, insufficient sun exposure, etc. [19]. Due to the effects of vitamin D on multiple organ systems, a vitamin D deficiency was reported to be associated with various dysfunctions, including osteoporosis and sarcopenia, physical function, cognitive function, and cardiovascular risk [20,21,22,23,24,25]. Visser et al. found that older individuals (with a mean age of 74 years) with a lower blood 25-hydroxyvitamin D level were significantly more likely to lose grip strength and muscle mass, and that 30 ng/mL may be a threshold for optimal muscle function [21]. Therefore, it was speculated that the high prevalence of vitamin D insufficiency in elderly nursing home residents might be caused by limited sun exposure and the sedentary lifestyle which are associated with the musculoskeletal system.

After 12 weeks, all groups showed higher blood hsCRP levels which is a predictor of cardiovascular diseases [12,13]. It was indicated that aging is associated with the development of a systemic state of low-grade chronic inflammation (inflammaging), and with progressive deterioration of metabolic function [26]. Furthermore, a high body fat percentage was also found in all groups (Table 3). Obesity, particularly visceral adiposity, is associated with chronic low-grade inflammation, as indicated by increased levels of the inflammatory markers CRP and interleukin (IL)-6 in the circulation of obese subjects [27]. Cesari et al. also indicated that CRP and IL-6 are positively associated with total fat mass and negatively associated with appendicular lean mass [28]. According to the results of hsCRP and body fat percentage in this study, it was considered that obesity-associated inflammation may play an important role in the age-related process that leads to sarcopenia. Inflammatory cytokines, such as IL-6 and TNF-α, should be measured in future studies.

### 4.2. Effects of Exercise on the Sarcopenic Index

In this study, it was found that calf circumference and gait speed significantly increased in the control group which received no supplementation (Table 3). It was reported that high-intensity resistance exercise training is a feasible and effective means of counteracting muscle weakness and physical frailty in very elderly people (87.1 ± 0.6 years of age); in contrast, multi-nutrient supplementation without concomitant exercise did not ameliorate muscle weakness or physical frailty [29]. It was also indicated that a high-intensity functional exercise program has positive long-term effects on balance, gait ability, and lower-limb strength for older persons dependent on activities of daily living (ADLs) [30]. However, Rosendahl et al. suggested that high-intensity exercise is not suitable for very old adults, as it might cause no obvious change in the appendicular skeletal muscle mass index [31].

### 4.3. Effects of Exercise Combined with Milk and Soy Milk Supplementation on the Sarcopenic Index

As described above, the amount of protein intake was increased from 1.3 to 1.5 g/kg BW in the milk group and from 1.4 to 1.6 g/kg BW in the soy milk group. Water et al. indicated that 1.2–1.5 g/kg BW protein intake is sufficient to prevent sarcopenia [32]. Thus, the dosage of protein supplementation used in this study might be adequate to prevent sarcopenia, but it is uncertain whether it could ameliorate sarcopenia or not. In this study, it was found that hand grip strength improved after milk and soy milk supplementation for 12 weeks (Table 3). In addition, calf circumference was also significantly elevated after soy milk supplementation for 12 weeks (Table 3). Kim et al. suggested that a combination of exercise and a leucine-rich essential amino acid mixture (3 g, twice a day for 3 months) may be effective in enhancing muscle strength and walking speed in sarcopenic women [33]. Furthermore, the AWGS 2019 update proposed separate algorithms for community versus hospital settings, both of which begin by screening either calf circumference (<34 cm in men and <33 cm in women) to facilitate earlier identification of people at risk for sarcopenia [34]. Several studies also demonstrated that calf circumference was negatively correlated with ADL scores; thus calf circumference is an important anthropometric indicator of physical function in the elderly [35,36].

Neither milk nor soy milk supplementation increased the appendicular skeletal muscle mass (Table 3). A previous study indicated that the daily consumption of low-fat fortified milk did not enhance the effects of resistance training on skeletal muscle size, strength, or function in healthy middle-aged and older men with adequate energy and nutrient intake [6]. Bonnefoy et al. also reported that nutritional supplements and exercise may improve muscle function, but had no significant results on skeletal muscle mass [37]. Singh et al. explained that age-related sarcopenia appeared largely confined to type II muscle fibers and the thigh muscle area; in addition, age and frailty may weaken the adaptive mechanisms loading the muscle mass [38]. Moreover, it was demonstrated that a significant reduction in dietary energy intake with supplements or non-energetic supplements failed to elevate the muscle mass in frail elderly with exercise training [29,39]. In this study, participants maintained similar energy intake levels except for milk (192 kcal/day) or soy milk (194.8 kcal/day) supplementation for 12 weeks (Table 5). Additionally, there was no change in the BMI during the experimental period (Table 3). Therefore, it was speculated that the lack of beneficial effects of exercise and supplements on muscle mass in this study might be related to the limited ability to load muscle mass instead of inadequate energy intake in old and very old individuals with sarcopenia.

As shown in Table 4, mild resistance exercise for 12 weeks improved the calf circumference and gait speed. Additionally, mild resistance exercise combined with milk or soy milk (400 mL/day) supplementation also increased hand grip and/or calf circumferences in very old nursing home residents with sarcopenia. Therefore, the combination of exercise and nutritional supplementation had beneficial effects on elevating muscle strength. However, the nutritional supplementation did not show the synergistic effect on the amelioration of sarcopenia in very old nursing home residents under mild resistance exercise. A possible reason is that all groups accepted the exercise, which might have reduced the influence of nutritional supplements in this study. In addition, the reduced ability to use available protein might be the other reason for the unapparent effects on improving muscle mass and strength after milk or soy milk supplementation in older people. A systematic review summarized that the biggest effect of any type of exercise intervention was on physical performance (gait speed, chair rising test, balance, etc.); however, the interactive effect of dietary supplementation on muscle function appears limited [40].

Loenneke et al. indicated that the consumption of 1–2 daily meals with protein content from 30 to 45 g may be an important strategy for increasing and/or maintaining lean body mass and muscle strength with aging [41]. In this study, the participants were provided with milk or soy milk twice after breakfast or before dinner within 30 min. Based on Table 5, the average protein intake per meal was around 25 or 26 g, which was elevated to 30 to 31 g after milk (5.2 g/200 mL) or soy milk (6.4 g/200 mL) supplementation. Therefore, the supplementation amount of protein has to be raised in future studies in order to significantly increase muscle mass or strength.

### 4.4. Effects of Exercise Combined with Milk and Soy Milk Supplementation on IGF-1

Blood IGF-1 level was significantly increased when supplemented with soy milk for 12 weeks (Table 2). Khalil et al. reported that supplementation with 40 g/day soy protein for 3 months increased serum IGF-I concentrations in men (27–84 y), compared with milk protein [42].

On the other hand, low IGF-I levels were associated with poor knee extensor muscle strength, slow walking speeds, and self-reported difficulty with mobility tasks in a study population including frail and healthy older women [9]. Moreover, Borst et al. indicated that increased IGF-1 may increase muscle strength that results from resistance training [43]. However, several studies demonstrated the weak association between blood IGF-1 levels and muscle strength in older adults compared to young adults [44,45,46,47]. In this study, the blood IGF-1 level was significantly elevated, whereas calf circumferences and hand grip were also improved after soy milk supplementation in very old individuals under mild resistance exercise (Table 2 and Table 4). It was difficult to clarify the improvement effects on muscle strength was due to the mild resistance exercise or soy milk because the supplemental amount of soy protein (12.8 g/day) in this study was less than that of the previous study [41]. This result must be confirmed in a future study.

### 4.5. Strengths and Limitations

This study has several strengths, among which we provide the first report to discuss sarcopenia in elderly nursing home residents in Taiwan. The study also used commercial products for nutritional supplementation so that they would be easy to apply in daily life. However, the study also has several limitations. First, the sample size was too small, and participants were from only two nursing homes. There is the issue of gender which is extremely unbalanced in three groups. When the females were dropped entirely, all results were the same as the results that included females. Second, participants with sarcopenia also had several chronic diseases which might have caused the results to be more complicated to interpret. Last, we are unable to discuss the favorable effect of nutritional supplementation on sarcopenia because all groups underwent exercise training.

## 5. Conclusions

Mild resistance exercise for 12 weeks improved the calf circumference and gait speed; in addition, mild resistance exercise combined with milk or soy milk (400 mL/day) supplementation also increased hand grip and/or calf circumferences in very old nursing home residents with sarcopenia. Therefore, the combination of exercise and nutritional supplementation had beneficial effects on elevating muscle strength; however, no obvious changes were found in the muscle mass of very old individuals with sarcopenia.

## Figures and Tables

**Table 1:** Basic characteristics of sarcopenic subjects ^1^.

Variable	Control (*n* = 12)	Milk (*n* = 12)	Soy Milk (*n* = 11)	*p* ^2^
	Mean ± SD or *n* (%)			
	Demographic data			
Gender				0.5528
Male, *n* (%)	9 (75)	11 (92)	9 (82)	
Female, *n* (%)	3 (25)	1 (8)	2 (18)	
Age (years)	84.67 ± 7.5	85.25 ± 5.38	85 ± 5.62	0.9741
	Medical history			
CVD (*n*)	9	9	8	0.9899
COPD (*n*)	5	6	6	0.8204
DM (*n*)	9	8	8	0.8970
CKD (*n*)	10	8	10	0.3271
Dementia (*n*)	11	11	8	0.3313

^1^ Values are expressed as the mean ± standard deviation (SD) or *n* (%). ^2^ *p* values were calculated among the three groups by a one-way ANOVA test and Chi-squared test. Abbreviations: CVD: cardiovascular disease; COPD: chronic obstructive pulmonary disease; DM: diabetes mellitus; CKD: chronic kidney disease.

**Table 2:** Effects of milk or soy milk combined with resistance exercise on clinical laboratory data of very old nursing home residents with sarcopenia ^1^.

Variable	Control (*n* = 12)		Milk (*n* = 12)		Soy Milk (*n* = 11)		*p* ^2^		
	Mean ± SD or *n* (%)						Time	Treatment	Time × Treatment
Liver function	Baseline	Week 12	Baseline	Week 12	Baseline	Week 12			
ALT (U/L)	12.2 ± 2.9	11.67 ± 3.5	15.3 ± 7.0	13.08 ± 4.8	10.8 ± 4.1	41.18 ± 99.0	0.3317	0.4293	0.2888
Kidney function									
Creatinine (mg/dL)	1.06 ± 0.35	1.0 ± 0.3	1.1 ± 0.4	1.07 ± 0.36	1.1 ± 0.3	1.05 ± 0.2	0.5524	0.8294	0.9873
Nutritional status									
Prealbumin (mg/dL)	19.3 ± 4.8	17.73 ± 6.3	20.4 ± 5.6	19.15 ± 3.8	22.3 ± 6.8	22.76 ± 6.2	0.5622	0.0527	0.8049
25-hydroxyvitamin D (ng/mL)	14.0 ± 7.0	11.85 ± 6.8	20.8 ± 8.6	17.96 ± 7.7	21.1 ± 7.8	17.13 ± 6.8	0.1003	0.606	0.9161
Inflammation index									
hsCRP (mg/dL)	0.7 ± 1.0	1.16 ± 1.64	0.7 ± 1.1	1.43 ± 2.2	1.3 ± 3.1	1.28 ± 1.79	0.3984	0.8115	0.7963
Insulin resistance index									
FBS (mg/dL)	83.7 ± 13.3	93 ± 20.6	93.3 ± 27.4	98 ± 40.6	108.6 ± 49.7	101.45 ± 22.96	0.7619	0.1993	0.6548
Insulin (mIU/L)	6.1 ± 4.9	6.9 ± 7.7	6.1 ± 4.2	6.1 ± 3.47	5.3 ± 2.2	8.0 ± 6.4	0.3628	0.9351	0.6632
HbA1c (%)	5.76 ± 0.43	5.68 ± 0.44	6.01 ± 1.24	5.93 ± 1.01	6.43 ± 1.28	6.08 ± 0.59	0.4341	0.1374	0.8410
HOMA-IR	1.31 ± 1.1	1.91 ± 3.05	1.37 ± 0.87	1.40 ± 0.73	1.39 ± 0.72	2.20 ± 2.09	0.2343	0.7048	0.7321
Hormonal factor									
IGF-1 (mg/dL)	73.3 ± 22.0	68.6 ± 28.1	92.7 ± 26.8	89.4 ± 34.4	103.0 ± 44.0	112.3 ± 49.7 ^#^	0.9587	0.003	0.7589

^1^ Values are expressed as the mean ± standard deviation (SD) or *n* (%). ^2^ *p* values were calculated among the three groups by a two-way ANOVA test and Chi-squared test. ^#^ Significant difference compared to control group at week 12 (*p* < 0.05). Abbreviations: ALT: alanine aminotransferase; hsCRP: high-sensitivity C-reactive protein; FBS: fasting blood sugar; HbA1c: hemoglobin A1c; HOMA-IR: homeostasis model assessment-insulin resistance index; IGF-1: insulin-like growth factor 1.

**Table 3:** Effects of milk or soy milk combined with resistance exercise on anthropometric data and sarcopenic index in very old nursing home residents with sarcopenia ^1^.

	Control (*n* = 12)			Milk (*n* = 12)			Soy Milk (*n* = 11)			*p* ^2^		
	Baseline	Week 6	Week 12	Baseline	Week 6	Week 12	Baseline	Week 6	Week 12	Time	Treatment	Time × Treatment
Anthropometric data												
BW (kg)	56.2 ± 8.6	54.7 ± 11.9	56.7 ± 8.4	58.3 ± 9.1	59.2 ± 9.1	59.7 ± 9	55.4 ± 8.7	55.2 ± 9.2	56.4 ± 9.1	0.9436	0.2345	0.9953
BMI (kg/m^2^)	22.1 ± 2.4	21.5 ± 4.1	22.3 ± 2.3	22.1 ± 3.2	22.4 ± 3.0	22.6 ± 3.1	22.6 ± 2.2	22.6 ± 2.7	23.1 ± 2.5	0.7484	0.5169	0.9893
Body fat (%)	30.6 ± 8.6	29.9 ± 9.1	31.7 ± 8.8	32.7 ± 5.6	29.3 ± 7.7	30.9 ± 6.	32.3 ± 6.5	32.4 ± 8.1	32.8 ± 7.7	0.7157	0.5801	0.9337
Sarcopenic index												
ASMI (kg/m^2^) ^3^	6.1 ± 0.6	6.0 ± 0.6	6.0 ± 0.7	6.2 ± 0.5	6.2 ± 0.61	6.0 ± 0.6	6.0 ± 0.7	6.1 ± 0.7	6.1 ± 0.7	0.8800	0.7997	0.9244
CC (cm)	30.8 ± 2.5	31.2 ± 2.2	31.5 ± 2.0 *	30.7 ± 2.6	30.6 ± 2.32	30.9 ± 2.5	31.3 ± 2.1	31.6 ± 2.0	32.3 ± 1.7 *	0.4843	0.1788	0.9784
HG (kg)	17.4 ± 5.3	17.6 ± 5.4	19.1 ± 5.6	18.7 ± 6.2	19.2 ± 5.62	21.2 ± 6.4 *	18.6 ± 6.8	19.5 ± 5.6	21.0 ± 6.1 *	0.2716	0.3985	0.9990
GS (m/s)	0.6 ± 0.3	0.6 ± 0.3	0.7 ± 0.3 *	0.4 ± 0.2	0.4 ± 0.22	0.4 ± 0.2	0.4 ± 0.2	0.4 ± 0.2	0.5 ± 0.3	0.5059	0.3504	0.9399

^1^ Values are expressed as the mean ± standard deviation (SD) or *n* (%). ^2^ *p* values represent differences among three groups by a two-way ANOVA test. ^3^ ASMI = ASM/height^2^ (kg/m^2^). * Significant difference compared to the baseline by a paired *t*-test within the group (*p* < 0.05). Abbreviations: BW: body weight; BMI: body-mass index; ASMI: appendicular skeletal muscle mass (ASM) index; CC: calf circumference; HG: hand grip; GS: gait speed.

**Table 4:** Effects of milk or soy milk combined with resistance exercise on the stage of sarcopenia in very old nursing home residents with sarcopenia ^1^.

Stage of Sarcopenia	Control (*n* = 12)		Milk (*n* = 12)		Soy Milk (*n* = 11)	
	Baseline	Week 12	Baseline	Week 12	Baseline	Week 12
Pre-sarcopenia (*n*, %)	0 (0%)	1 (8%)	0 (0%)	0	0	1 (9%)
Sarcopenia (*n*, %)	3 (25%)	9 (75%)	3 (25%)	4 (33%)	2 (18%)	4 (36%)
Severe sarcopenia (*n*, %)	9 (75%)	2 (17%)	9 (75%)	8 (67%)	9 (82%)	6 (55%)
*p* ^2^	0.0146		NA		0.322	

^1^ The classification is according to The European Working Group on Sarcopenia in Older People (EWGSOP). ^2^ *p* values represent the differences between the baseline and week 12 by a Chi-squared test. Abbreviation: NA: not applicable.

**Table 5:** Effects of milk or soy milk combined with resistance exercise on the dietary intake of very old nursing home residents with sarcopenia ^1^.

	Control (*n* = 12)		Milk (*n* = 11)		Soy Milk (*n* = 12)		*p* ^2^		
Nutrients	Baseline	Week 12	Baseline	Week 12	Baseline	Week 12	Time	Treatment	Time × Treatment
Energy (kcal/day)	1677 ± 319	1714 ± 252	1857 ± 195	1881 ± 204	1845 ± 219	1898 ± 247	0.5176	0.0235	0.9796
CHO (g/day)	225 ± 52	225 ± 43	256 ± 35	241 ± 41	251 ± 44	247 ± 30	0.4631	0.1158	0.8612
Protein (g/day)	70.3 ± 11.9	74.7 ± 15.4	76.4 ± 9.5	77.3 ± 3.2	76.1 ± 7.9	77.7 ± 7.9	0.3453	0.2413	0.8230
Fat (g/day)	54.8 ± 10.7	55.8 ± 11.2	59.5 ± 8.8	61.5 ± 11.6	60.6 ± 7.9	61.4 ± 7.6	0.5889	0.0950	0.9750

^1^ Data were calculated based on 24 h dietary recall including one weekday and two weekend days. Data are expressed as the mean ± standard deviation (SD). ^2^ *p* values represent the differences among the three groups by a two-way ANOVA. *p* values were calculated from the mean difference between the baseline and week 12 within the same group by a paired *t*-test. Abbreviation: CHO: carbohydrates.
